# Anti-inflammatory effect of bee pollen ethanol extract from *Cistus *sp. of Spanish on carrageenan-induced rat hind paw edema

**DOI:** 10.1186/1472-6882-10-30

**Published:** 2010-06-23

**Authors:** Hiroe Maruyama, Takashi Sakamoto, Yoko Araki, Hideaki Hara

**Affiliations:** 1 Nagaragawa Research Center, API Co. Ltd. 692-3 Nagarayamasaki, Gifu 502-0071, Japan; 2 Department of Biofunctional Evaluation, Molecular Pharmacology, Gifu Pharmaceutical University, 1-25-4 Daigaku-nishi, Gifu 501-1196, Japan

## Abstract

**Background:**

Bee pollen, a honeybee product, is the feed for honeybees prepared themselves by pollens collecting from plants and has been consumed as a perfect food in Europe, because it is nutritionally well balanced. In this study, we aimed to investigate the anti-inflammatory effect of bee pollen from *Cistus *sp. of Spanish origin by a method of carrageenan-induced paw edema in rats, and to investigate the mechanism of anti-inflammatory action and also to elucidate components involved in bee pollen extracted with ethanol.

**Methods:**

The bee pollen bulk, its water extract and its ethanol extract were administered orally to rats. One hour later, paw edema was produced by injecting of 1% solution of carrageenan, and paw volume was measured before and after carrageenan injection up to 5 h. The ethanol extract and water extract were measured COX-1 and COX-2 inhibitory activities using COX inhibitor screening assay kit, and were compared for the inhibition of NO production in LPS-stimulated RAW 264.7 cells. The constituents of bee pollen were purified from the ethanol extract subjected to silica gel or LH-20 column chromatography. Each column chromatography fractions were further purified by repeated ODS or silica gel column chromatography.

**Results:**

The bee pollen bulk mildly suppressed the carrageenan-induced paw edema and the water extract showed almost no inhibitory activity, but the ethanol extract showed relatively strong inhibition of paw edema. The ethanol extract inhibited the NO production and COX-2 but not COX-1 activity, but the water extract did not affect the NO production or COX activities. Flavonoids were isolated and purified from the ethanol extract of bee pollen, and identified at least five flavonoids and their glycosides.

**Conclusions:**

It is suggested that the ethanol extract of bee pollen show a potent anti-inflammatory activity and its effect acts *via *the inhibition of NO production, besides the inhibitory activity of COX-2. Some flavonoids included in bee pollen may partly participate in some of the anti-inflammatory action. The bee pollen would be beneficial not only as a dietary supplement but also as a functional food.

## Background

There are roughly two groups of pollen materials. One group is made by honeybees and the other is directly collected from the flower of plants. The former group is the feed for honeybees prepared by mixing honey with pollens collected from plants and called bee pollen or pollen ball. Bee pollen is collected by beekeepers with the use of a screen over hive openings designed specifically to let the bees pass while squeezing pollen from their hind legs and pollen sacs, and has its own specificity, mainly linked to the floral species or cultivars [1].

Bee pollen is rich in protein, particularly free amino acids, and also abounds with carbohydrate, lipid, vitamins and minerals [2,3]. In addition, bee pollen contains minor components, such as flavonoids and phenolic compounds [4,5]. Bee pollen, which is nutritionally well balanced, has been consumed as a perfect food in Europe and the U.S. for a long time.

Although there have been many studies on the functionality of pollens directly collected from plants, there have not been many reports on the functionality of bee pollen. There have been some reports on bee pollen but they provided extremely few data by source plant. It has been reported that bee pollen from *Cistus *sp. of Spanish origin prevents osteoporosis by increasing bone mass and exhibits antiallergic action [6-10]. In addition, bee pollen has been reported to show antioxidant and radical scavenging activities [11], and recently, Akkol et al. have reported that antinociceptive, anti-inflammatory, gastroprotective and antioxidant effects of pure honey and honey-bee pollen mix formulation were evaluated comparatively [12].

Concerning pollens directly collected from plants, their effect on prostatitis in men and anti-inflammatory effect in animal experiments have been confirmed though their active components for anti-inflammatory action have not been identified [13,14]. On the other hand, phenolic and flavonoid components of honey-bee pollen mix involved in anti-inflammatory action have been reported by Akkol et al. [12].

In this study, we aimed to investigate the anti-inflammatory effect of bee pollen from *Cistus *sp. of Spanish origin by a method of carrageenan-induced paw edema in rats, and to investigate the mechanism of anti-inflammatory action and also to elucidate components involved in bee pollen extracted with ethanol.

## Methods

### Materials

Bee Pollen from *Cistus *sp. of Spanish origin and Bee Pollen from *Brassica *sp. of China origin were obtained from Api Co., Ltd.

The following drugs and chemicals were purchased and used: λ-carrageenan, indomethacin (Wako Pure Chemical Industries, Ltd., Osaka, Japan), lipopolysaccaride (LPS), Griess reagent, DMEM and other cell culture reagents including FBS (Sigma Chemical Co., St. Louis, MO, U.S.A.).

### Particle size distribution

Particle size distribution of bee pollen from *Cistus *sp. and *Brassica *sp. were measured by Coulter counter multisizer TM3. (Beckman Coulter, Miami, FL, U.S.A.) [15,16]. A Coulter counter with 100 μm aperture (particle size; 2-60 μm, counting particles; 50000) was used for particle analysis. Before the analysis, each sample was diluted with electrolyte solution (ISOTON II) to the appropriate concentration of particles and sonicated for 5 min. The obtained distribution was a volume distribution.

### Bee pollen extracts

Each 200 g of bee pollen (BP) from *Cistus *sp. of Spanish origin was extracted with water (Water BP) or 95% ethanol (EtOH BP), and stirred for 4 h at 60°C or stirred for 16 h at room temperature, respectively. The filtrate was taken to dryness and powdered. The yields were 40.9% (Water BP, 81.8 g) and 17.1% (EtOH BP, 34.2 g).

### Animals

Male Wistar rats of 6 weeks old were obtained from SLC Japan, Inc. (Shizuoka, Japan). Animals were housed in steel cages in a room kept at 23°C with a 12-h light-dark cycle (lights on 8:00-20:00), and fed a laboratory diet (CE-II, CLEA Japan, Inc., Tokyo, Japan). Water was freely available. The animal experimental protocol was approved prior to its execution by our in-house committee in accordance with the National Institute of Health Guideline for the Care and Use of Laboratory Animals, the Japanese Government Animal Protection and Management Law Number 105 and the Japanese Government Notification on Feeding and Safekeeping of Animals Number 6.

### Carrageenan-induced rat hind paw edema

Male Wistar rats were fasted for 16 h and used in groups of 4, each containing 7 individuals. Bee Pollen (300 mg/kg), Water BP (300 mg/kg), EtOH BP (100 and 300 mg/kg) and indomethacin (30 mg/kg) were suspended in a 5% solution of gum Arabic (vehicle) and were administered orally. One hour later, paw edema was produced by injecting 100 μl of 1% solution of carrageenan in saline into the left hind paw. Paw volume was measured before and after carrageenan injection up to 5 h, using a water displacement plethysmometry (plethysmometer MK-550, Muromachi Kikai Co., Tokyo, Japan). The swelling ratio (% swelling) was expressed as the percentage of the increase in paw volume before carrageenan injection [17].

### Cox inhibitory activity

EtOH BP and Water BP were measured COX inhibitory activities of COX inhibitor screening assay kit (Cayman Chemical Company, Ann Arbor, MI, U.S.A) [18-20]. The COX inhibitor screening assay directly measures PGF_2α _produced by SnCl_2 _reduction of COX-derived PGH_2 _produced in the COX reaction. All procedures were performed as indicated in the assay kit instructions. Briefly, the reaction buffer (Tris-HCl buffer, pH 8.0, containing 5 mM EDTA and 2 mM phenol) and heme were placed in test tubes [18]. The sample and COX-1 or COX-2 were added to the test tubes and pre-incubated for 10 min at 37°C. After the substrate arachidonic acid was added, the test tubes were incubated for 2 min at 37°C. The concentration of PGF_2α _was measured using the enzyme immunoassay (EIA) of the same kit. EtOH BP and Water BP samples were added to the test tubes at final concentrations of 150, 50, 16.7 and 5.6 μg/ml within the range of three time dosage ratio. All samples were added as dimethyl sulfoxide (DMSO) solutions to assay solutions, and all determinations were performed in triplicate (N = 3). Values for 50% inhibitory concentration (IC_50_) were obtained by linear regression analysis.

### RAW 264.7 cell culture and measurement of NO production

RAW 264.7 cells obtained from the American Type Culture Collection were cultured with DMEM supplemented with 10% FBS and 1% antibiotics under 5% CO_2 _at 37°C and activated with LPS according to the previously described procedures [21,22]. In brief, the cells were plated in 96-well plates (2 × 10^5 ^cells/well). After pre-incubation for 2 h, the test samples including EtOH BP or Water BP and LPS (1 μg/ml) were added and the cells were incubated for 24 h. Test samples were dissolved in DMSO on the day of the experiment and diluted with serum-free DMEM into appropriate concentrations. Final concentration of DMSO was adjusted to 0.1% (v/v). Control groups also received the same amount of DMSO. Cell viability was assessed with MTT assay. For a determination of NO concentration, the amount of nitrite in the cell culture supernatant measured by using Griess reagents. The cell culture medium was mixed with Griess reagent and incubated for 10 min at room temperature. The optical density was measured with a microplate reader (μQuant, BIO-TEK instruments, Inc., VT, U.S.A.). at 550 nm. Nitrite was quantified by using sodium nitrate as a standard. Values for 50% inhibitory concentration (IC_50_) were obtained by linear regression analysis.

### Fractionation of bee pollen and Identification of flavonoids

Bee pollen from *Cistus *sp. of Spanish origin was successively extracted with water two times, and then the residue was extracted two times with hexane and two times with ethanol, subsequently. These procedures were performed at room temperature. Evaporation of the solvents in vacuo gave water, hexane and ethanol extracts.

**Figure 1:**
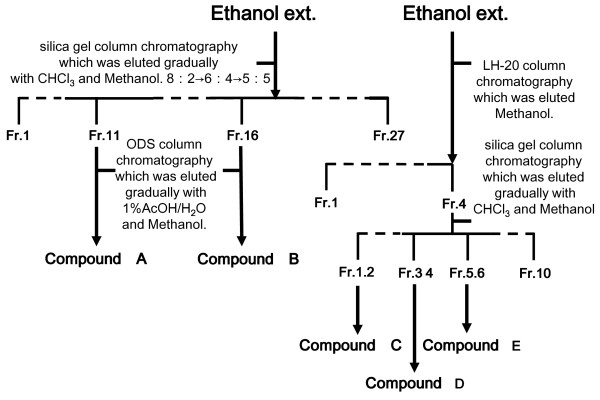
**Flowchart of the isolation process for the ethanol extract of bee pollen**. Ethanol extract of bee pollen was subjected to silica gel column chromatography eluted with CHCl_3 _and methanol or Sephadex™ LH-20 column chromatography eluted with methanol. Each column chromatography fractions were further purified by repeated ODS or silica gel column chromatography, finally yielding purified 5 compounds.

The process of isolation is shown in Figure 1. Ethanol extract (31 g, Figure 1 left side) was further purified using the silica gel column chromatography CHCl_3_-MeOH system (gradually replaced with MeOH 20-50%) [23,24]. Fractions were collected and their composition monitored by TLC (solvents: CHCl_3_-MeOH = 7:3) and those with similar TLC profiles were combined into 27 major fractions denoted as Fr. 1 to Fr. 27. Each fraction was collected and dried. Fraction 11 and 16 was further purified by ODS column chromatography methanol-1% acetic acid system, finally yielding purified compound A (4.9 mg, kaempferol-3-glucoside) and B (20 mg, quercetin-7-rhamnoside). And, ethanol extract (2 g, Figure 1 right side) was applied to Sephadex™ LH-20 (Amersham Biosciences, Piscataway, NJ, U.S.A.) column chromatography (*ϕ *25 mm × 270 mm) eluted with methanol. Fractions (each 5 ml) were collected and their composition monitored by TLC (solvents: CHCl_3_-MeOH = 7:3) and those with similar TLC profiles were combined into 4 major fractions denoted as Fr. 1 to Fr. 4. Each fraction was collected and dried. Fraction 4 was further purified by silica gel column chromatography CHCl_3_-MeOH system, and finally yielding purified compound C (isorhamnetin), compound D (kaempferol), and compound E (quercetin). Flavonoids were identified by ^1^H-NMR, ^13^C-NMR and 2D-NMR (Varian, MERCURY plus 300 MHz; Varian Technologies Japan Ltd., Osaka, Japan) spectroscopic methods. The chemical shift values are reported in ppm (δ), and the coupling constants (J) are reported in Hz. Electro spray ionization mass spectra (ESI-MS) were obtained on a LCQ mass spectrometer (Thermo Fisher Scientific) and wherever possible by chromatographic TLC and high-performance liquid chromatography (HPLC) comparisons with authentic markers. An HPLC system equipped with a model 2695 or 996-Photodiodearray detector was used (Waters, Osaka, Japan). Chromatography was performed at 40°C using a CAPCELLPAK ACR column (Shiseido, Tokyo, Japan). Elution was performed in stepwise gradient mode. A: H_2_O containing 1% acetic acid. B: CH_3_CN containing 1% acetic acid [B concentration 20→80% (0→60 min, 80% hold (60-70 min)] at a flow rate of 1.0 ml/min.

### Data Analysis

In animal experiments, the results are expressed as means and standard deviations (S.D.). The significance of the differences was analyzed by Dunnett's multiple-range test.

## Results

### Particle size distribution

**Figure 2:**
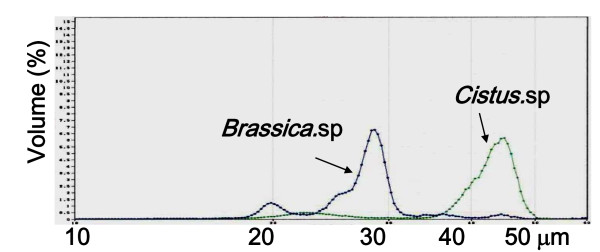
**Particle size distribution of bee pollen from *Cistus *sp. and *Brassica *sp. determined by Coulter counter**. A Coulter counter with 100 μm aperture (particle size; 2-60 μm, particle counting; 50000) was used for particle analysis. Before the analysis, each sample was diluted with electrolyte solution to the appropriate concentration of particles and sonicated for 5 min.

Figure 2 shows the particle size distributions of bee pollen from *Cistus *sp. and *Brassica *sp. The two kinds of bee pollens were clearly differed in distribution pattern. Bee pollen from *Cistus *sp. had a main peak in a range of 40-50 μm. In contrast, the one from *Brassica *sp. had a main peak near 28 μm and a small peak near 20 μm. Therefore, bee pollen from *Cistus *sp. showed more homogenous in flower composition.

### Anti-inflammatory effects of bee pollen on carrageenan-induced edema in rat hind paws

**Figure 3:**
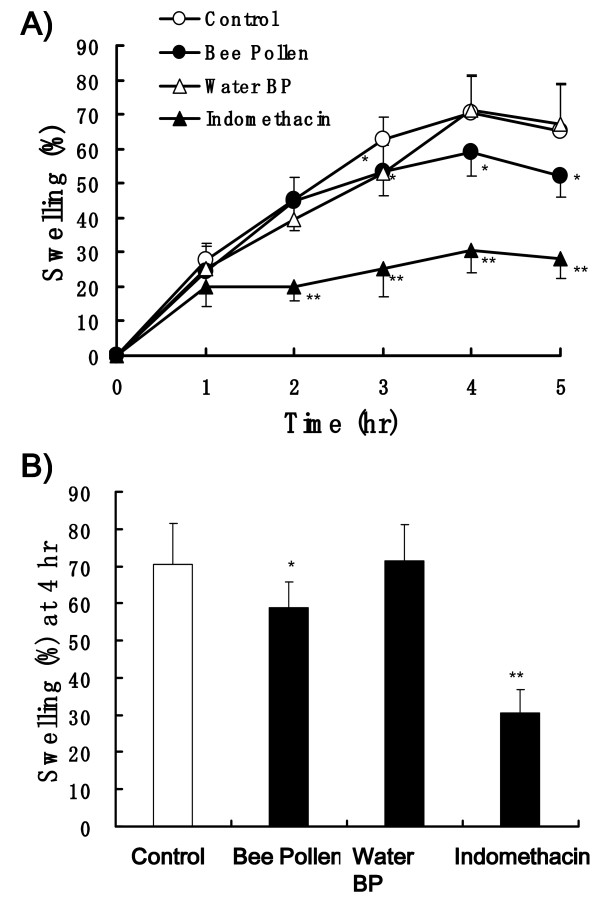
**Effects of bee pollen and indomethacin on carrageenan-induced paw edema in rats**. Bee pollen (300 mg/kg), Water BP (300 mg/kg) and indomethacin (30 mg/kg) were administered orally 60 min before carrageenan injection. (A) Time course of paw edema. (B) The swelling % measured 4 h after carrageenan injection. Values represent the mean ± SD, *n *= 7. *P < 0.05, **P < 0.01 *vs*. control (Dunnett's multiple-range test).

**Figure 4:**
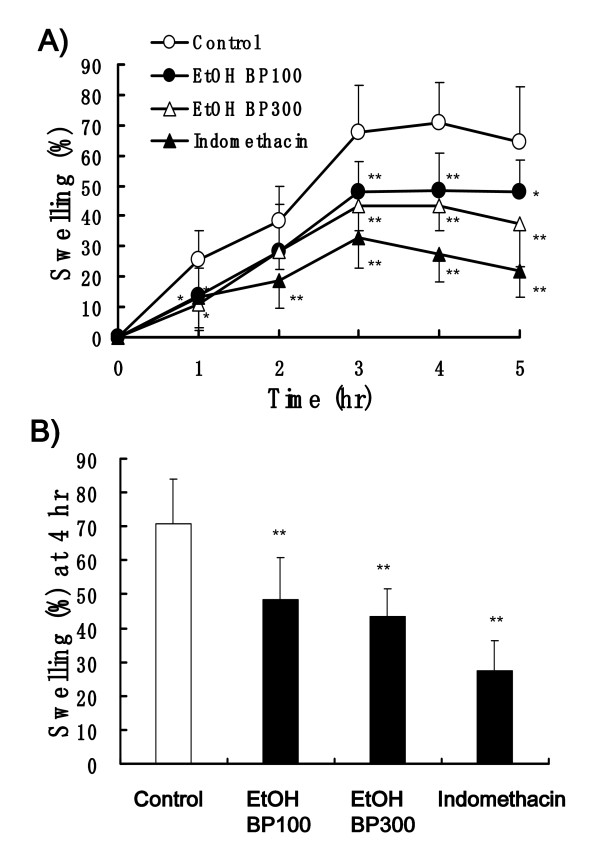
**Effects of bee pollen and indomethacin on carrageenan-induced paw edema in rats**. EtOH BP (100 and 300 mg/kg) and indomethacin (30 mg/kg) were administered orally 60 min before carrageenan injection. (A) Time course of paw edema. (B) The swelling % measured 4 h after carrageenan injection. Values represent the mean ± SD, *n *= 7. *P < 0.05, **P < 0.01 *vs*. control (Dunnett's multiple-range test).

After 100 μl of 1% carrageenan was injected into the hind paw, the paw edema in the control rats increased along with the time course and the peak edema was showed at 4 h (Figure 3A and Figure 4A). Mean % swelling of control group rats was 70.4 and 70.7% at 4 h (Figure 3B and Figure 4B). Bee Pollen from *Cistus *sp. at the dose of 300 mg/kg slightly, but significantly decreased the edema and 30 mg/kg dose of indomethacin significantly decreased the edema, 59.0 and 30.5% of swelling, respectively. Water BP at the dose of 300 m/kg almost did not affect the paw edema (Figure 3A and 3B).

On the other hand, EtOH BP (100 and 300 mg/kg) showed relatively strong and significantly inhibition of carrageenan-induced edema. Mean % swelling of 100 and 300 mg/kg of EtOH BP and indomethacin (30 mg/kg) was 48.4, 43.5 and 27.3%, respectively (Figure 4A and 4B).

### Inhibition of the COX-1 and COX-2 activities

**Figure 5:**
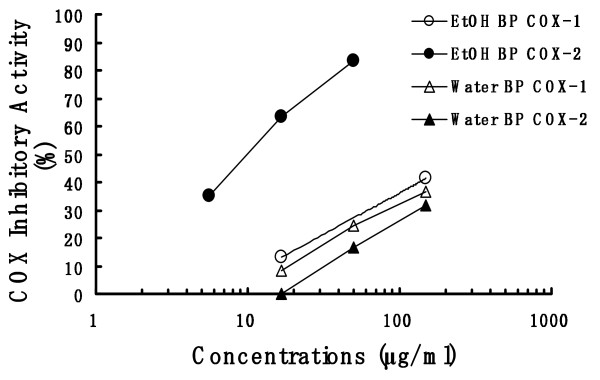
**The inhibitory effect of bee pollen on COX-1 and COX-2**. Each extracts of bee pollen (EtOH BP and Water BP) were measured COX inhibitory activities of COX inhibitor screening assay kit. The COX inhibitory activity (%) was represented as inhibition % of control (solvent-treated samples).

EtOH BP and Water BP were measured COX inhibitory activities using COX inhibitor screening assay kit. The inhibitory effects on COX-1 and COX-2 of EtOH BP showed concentration-dependently (Figure 5). As regards the COX-2 experiment, all EtOH BP groups showed significant decrease as compared with the control value (5.6 μg/ml; p < 0.05, 16.7 and 50 μg/ml; p < 0.01). The IC_50 _values for COX-1 and COX-2 were >150 μg/ml and 10.3 μg/ml, respectively. However, the inhibition of COX-2 activity of Water BP was lower than that of EtOH BP, and no differences in the COX-1 and COX-2 experiment were observed between Water BP group and the control group. The IC_50 _values for both COX-1 and COX-2 were >150 μg/ml.

### Effect of bee pollen extracts on NO production

**Figure 6:**
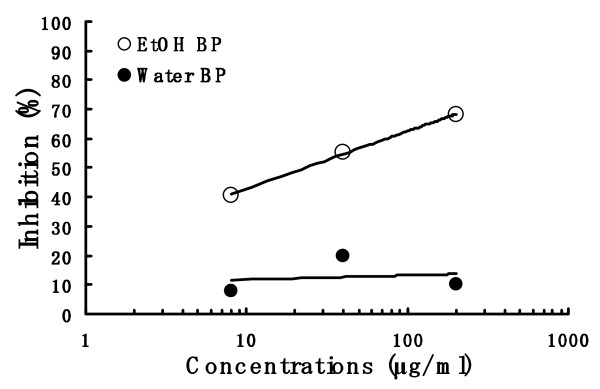
**Effect of bee pollen on NO production in LPS-stimulated RAW 264.7 cells**. The production of NO was detected and analyzed using Griess reagent. RAW 264.7 cells were incubated for 24 h at 37°C in medium containing three different concentrations of the bee pollen extract of each EtOH BP and Water BP and LPS (1 μg/ml). Results were expressed as an inhibition % of control (solvent-treated samples).

RAW 264.7 cells were incubated for 24 h in medium containing three different concentrations of the bee pollen extract of each EtOH BP and Water BP and LPS (1 μg/ml). EtOH BP inhibited concentration-dependently the LPS-induced production of nitrite (Figure 6) and its IC_50 _value was 23.2 μg/ml. However, Water BP did not inhibit the NO production. The cytotoxic effect of EtOH BP and Water BP were not observed under same experimental condition (data not shown).

### Isolation and identification of flavonoids

The results of the present study indicated that the anti-inflammatory effect of ethanol extract of bee pollen from *Cistus *sp. of Spanish origin was stronger than those of bulk and water extract of bee pollen. Therefore, we isolated the flavonoid components in the ethanol extract of bee pollen. The purity of these flavonoids, yielding compound A (180 mg), compound B (5 mg), compound C (3 mg), compound D (5 mg) and compound E (10 mg) was confirmed to be 90% or more by HPLC analysis.

The NMR data of compounds A to E are as follows:

#### Compound A: Kaempferol-3-glucoside

^1^H-NMR (DMSO-d_6_) δ: 3.1-3.8 (m, -glucose), 5.2 (1H, d, J = 7 Hz, glucose), 5.7 (1H, d, J = 2.5 Hz, H-6), 5.8 (1H, d, J = 2.5 Hz, H-8), 6.9 (2H, d, J = 9 Hz, H-3',5'), 8.06 (2H, d, J = 9 Hz, H-2', 6').

#### Compound B: Quercetin-7-rhamnoside

^1^H-NMR (DMSO-d_6_) δ: 1.2 (3H, m, J = 6Hz, CH_3_), 5.5 (1H, d, J = 2.5Hz, rhamnose), 6.2 (1H, d, J = 2.5 Hz, H-6), 6.2 (1H, d, J = 2.5 Hz, H-6), 6.4 (1H, d, J = 2.5 Hz, H-8), 6.8 (1H, d, J = 8.5 Hz, H-5'), 7.3 (1H, dd, H-6'), 7.4(1H, d, J = 8.5 Hz, H-2').

#### Compound C: Isorhamnetin

^1^H-NMR (DMSO-d_6_) δ: 3.84 (3H, s, -OMe), 6.19 (1H d, H-6), 6.47 (1H, d, H-8), 6.99 (1H, d, J = 8.5 Hz, H-5'), 7.68 (1H, dd, J = 8.5, 2.1 Hz, H-2', 6'), 7.75 (2H, dd, J = 2.1 Hz, H-2'), 12.47 (1H, s, -OH).

#### Compound D: Kaempferol

^1^H-NMR (DMSO-d_6_) δ: 6.18 (1H, brs, H-6), 6.39 (1H, brs, H-8), 6.92 (2H, d, J = 8.5 Hz, H-3',5'), 8.08 (2H, d, J = 8.9 Hz, H-2',6'),12.49 (1H, s, -OH).

#### Compound E: Quercetin

^1^H-NMR (DMSO-d_6_) δ: 6.18 (1H, d, J = 2.5 Hz, H-8), 6.40 (1H, d, J = 2.5 Hz, H-6), 6.86 (1H, d, J = 8.5 Hz, H-5'), 7.52 (1H, q, J = 8.5 Hz, H-6'), 7.67 (1H, d, J = 8.5 Hz, H-2').

**Figure 7:**
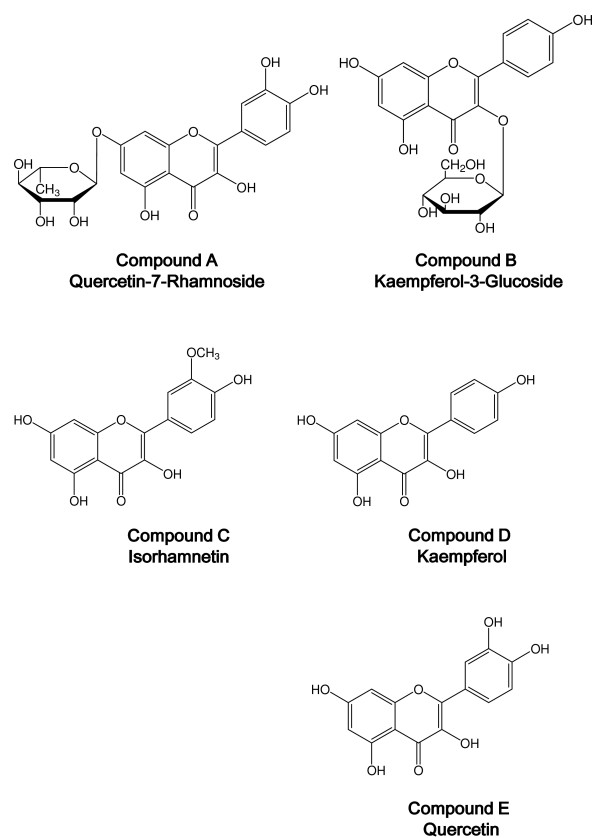
**Chemical structures of 5 compounds from the ethanol extract of bee pollen**. Compound A: Quercetin-7-Rhamnoside; Compound B: Kaempferol-3-Glucoside; Compound C: Isorhamnetin; Compound D: Kaempferol; Compound E: Quercetin

These compounds were identified by HPLC and NMR, which showed that the spectra were consistent with those reported previously [25-27]. The structures of these five compounds are shown in Figure 7.

## Discussion

Bee pollen contains sugars derived from honeybees and lipid complex from pollens. Because bee pollen is usually dried completely, pollen particles are sticking to one another. We added two kinds of bee pollens, one from a *Cistus *sp. of Spanish origin and the other from a *Brassica *sp. of China origin, to an electrolytic solution for dispersion and measured particle size distribution with a Coulter counter. The two kinds of bee pollens were clearly differed in distribution pattern, and the one from *Cistus *sp. showed more homogenous in flower composition. Therefore, the bee pollen from the *Cistus *sp. of Spanish origin was used in this study, because it was more homogenous in flower (pollen composition) and most frequently consumed as food in Japan.

We investigated the anti-inflammatory effect of bee pollen bulk, its water extract and its ethanol extract by a method of carrageenan-induced paw edema in rats. The bee pollen bulk mildly suppressed paw edema and the water extract showed almost no inhibitory activity (Figure 3), but the ethanol extract showed relatively strong inhibition of carrageenan-induced paw edema (Figure 4). These results suggest that ethanol extract of bee pollen show a potent anti-inflammatory activity.

The anti-inflammatory drugs, such as aspirin and indomethacin, have been found to inhibit prostaglandin production to cause anti-inflammatory action in carrageenan-induced paw edema [28,29]. Recently, it has also been reported that NO produced by i-NOS is involved in the inflammatory response on paw edema [17,30]. Therefore, the ethanol extract and water extract from the bee pollen were compared for the inhibition of NO production in RAW 264.7 cells and the inhibition of COX activity in vitro.

The water extract and ethanol extract little inhibited COX-1, but the ethanol extract inhibited COX-2 relatively strongly (Figure 5). The production of prostaglandins such as PGE_2 _and/or PGI_2 _is facilitated through COX-2 activity in inflammatory response [29]. These data indicate that the mechanism of anti-inflammatory action of the ethanol extract of bee pollen involves the inhibitory activity of COX-2.

In the experiment of NO production in RAW 264.7 cells, the water extract of bee pollen showed no suppression of NO production, but the ethanol extract inhibited NO production concentration-dependently (Figure 6). From the results, it is suggested that the anti-inflammatory effect of the ethanol extract on carrageenan-induced paw edema acts *via *the inhibition of NO production, besides the inhibitory activity of COX-2.

Flavonoids were isolated and purified from the ethanol extract fraction of the bee pollen from a *Cistus *sp. to identify at least five flavonoids and flavonoid glycosides: quercetin-7-rhamnoside, kaempferol-3-glucoside, isorhamnetin, kaempferol and quercetin. The Spanish bee pollen has been reported to contain their aglycones [4].

Various flavonoid derivatives including quercetin have been reported to inhibit the activity of arachidonic acid metabolizing enzymes (phospholipase A_2_, cycloxygenase and lipoxygenase) [31]. And it have been shown that certain flavonoids express their some of anti-inflammatory actions by modulation of proinflammatory gene expression, such as COX-2, i-NOS and several pivotal cytokines [32]. Hence, some flavonoids included in bee pollen may partly participate in some of the anti-inflammatory action, however because the inhibitory activities of COX-1 and COX-2 of the ethanol extract of bee pollen are relatively selective, other unknown substances may also be involved. The total phenolic contents as well as flavonoid of honey-bee pollen mix have been estimated in the in vivo activity assessment by Akkol et al. [12]. Further studies are needed to clarify the possible mechanism for flavonoids and other constituents.

## Conclusions

These findings presented here indicate that the ethanol extract of bee pollen show a potent anti-inflammatory activity and its effect acts *via *the inhibition of NO production, besides the inhibitory activity of COX-2. Some flavonoids included in bee pollen may partly participate in some of the anti-inflammatory action. The bee pollen would be beneficial not only as a dietary supplement but also as a functional food.

## Competing interests

The authors declare that they have no competing interests.

## Authors' contributions

HM, TS-Collection, analysis, and interpretation of the data; YA, HH-Design of the study management; HM, TS, YA, HH-Preparation, review, or approval of the manuscript. All authors read and approved the final manuscript.

## Pre-publication history

The pre-publication history for this paper can be accessed here:

http://www.biomedcentral.com/1472-6882/10/30/prepub
